# Prenatal Intestinal Obstruction Affects the Myenteric Plexus and Causes Functional Bowel Impairment in Fetal Rat Experimental Model of Intestinal Atresia

**DOI:** 10.1371/journal.pone.0062292

**Published:** 2013-05-08

**Authors:** Naziha Khen-Dunlop, Sabine Sarnacki, Anais Victor, Celine Grosos, Sandrine Menard, Rodolphe Soret, Nicolas Goudin, Maud Pousset, Frederique Sauvat, Yann Revillon, Nadine Cerf-Bensussan, Michel Neunlist

**Affiliations:** 1 INSERM U989, Paris, France; 2 AP-HP, Hôpital Necker-Enfants Malades, Service de Chirurgie Pediatrique, Paris, France; 3 Université Paris Descartes, Sorbonne Paris Cité, Paris, France; 4 INSERM U913, Nantes, France; 5 CHU Nantes, Hôtel Dieu, Institut des Maladies de l’Appareil Digestif, Nantes, France; 6 Université de Nantes, Faculté de Médecine, Nantes, France; 7 AP-HP, Hôpital Necker-Enfants Malades, Plateforme d’Imagerie, Paris, France; 8 AP-HP, Hôpital Necker-Enfants Malades, Département de Biostatistiques, Paris, France; Hôpital Robert Debré, France

## Abstract

**Background:**

Intestinal atresia is a rare congenital disorder with an incidence of 3/10 000 birth. About one-third of patients have severe intestinal dysfunction after surgical repair. We examined whether prenatal gastrointestinal obstruction might effect on the myenteric plexus and account for subsequent functional disorders.

**Methodology/Principal Findings:**

We studied a rat model of surgically induced antenatal atresia, comparing intestinal samples from both sides of the obstruction and with healthy rat pups controls. Whole-mount preparations of the myenteric plexus were stained for choline acetyltransferase (ChAT) and nitric oxide synthase (nNOS). Quantitative reverse transcription PCR was used to analyze mRNAs for inflammatory markers. Functional motility and permeability analyses were performed in vitro. Phenotypic studies were also performed in 8 newborns with intestinal atresia. In the experimental model, the proportion of nNOS-immunoreactive neurons was similar in proximal and distal segments (6.7±4.6% vs 5.6±4.2%, p = 0.25), but proximal segments contained a higher proportion of ChAT-immunoreactive neurons (13.2±6.2% vs 7.5±4.3%, p = 0.005). Phenotypic changes were associated with a 100-fold lower concentration-dependent contractile response to carbachol and a 1.6-fold higher EFS-induced contractile response in proximal compared to distal segments. Transcellular (p = 0.002) but not paracellular permeability was increased. Comparison with controls showed that modifications involved not only proximal but also distal segments. Phenotypic studies in human atresia confirmed the changes in ChAT expression.

**Conclusion:**

Experimental atresia in fetal rat induces differential myenteric plexus phenotypical as well as functional changes (motility and permeability) between the two sides of the obstruction. Delineating these changes might help to identify markers predictive of motility dysfunction and to define guidelines for post-surgical care.

## Introduction

Intestinal atresia is a common congenital gut disorder characterized by the interruption of intestinal continuity. Its prevalence is about 3/10 000 births (1). The diagnosis is generally made during the second or third trimester, based on ultrasound detection of bowel dilation [1]. In jejuno-ileal atresia, the obstruction is commonly related to a fetal vascular event secondary to mesenteric ischemia, intestinal volvulus, intussusception or strangulation [2], [3]. Genetic factors are not involved, except in rare syndromic forms [4], [5]. Surgical repair is necessary soon after birth, because of the obstructive consequences, and usually consists of gut resection-anastomosis. However, surgical repair is followed by severe intestinal dysmotility in about one-third of cases, necessitating prolonged parenteral nutrition [6]. These neonates are exposed to sepsis linked to gut bacterial translocation and parenteral nutrition-induced liver disease, and require lengthy and costly inpatient management.

Initiation and regulation of small-bowel motility depends on normal functioning of several structures, and particularly the enteric nervous system (ENS). The ENS is an integrative network made up of neurons and glial cells derived from the neural crest. It is located all along the gut and regulates intestinal peristalsis and secretion [7]. It comprises the myenteric plexus, which primarily controls motor functions, and the submucosal plexus, which regulates electrolyte transport, intestinal barrier permeability and mucosal blood flow [7], [8]. A specific neurochemical code of neurons (a combination of neuromediators and enzymes) is often associated with a specific neuronal function (muscle motorneurons, secretomotorneurons, sensory neurons) [9]. Therefore, ENS disorders, ranging from changes in neurochemical coding to neuronal cell death, could be directly responsible for the intestinal dysfunction associated with some gastrointestinal disorders [10].

Although it is well recognized that atresia leads to morphological alterations in proximal and distal segments, there is so far little evidence that ENS alterations participate in the motility disorders observed after surgical treatment of congenital intestinal atresia. Only scattered and descriptive assessments of the ENS are available. Current data points largely to alterations in the proximal dilated segment but fail to show major alterations in the distal segment. In particular, decreases in NADPH-diaphorase, PGP9.5 and VIP nerve density have been described upstream of the atresia [11]–[13], and reduced acetylcholine esterase staining has been observed downstream [14], [15]. In a preliminary semi-quantitative study of human atresia, we observed changes in the architecture of the myenteric plexus, but ENS neurochemical coding was not examined [16].

In the present study, using an experimental rat model of prenatal intestinal atresia [17], we aimed to characterize 1) neuroplastic changes in the ENS from both parts of the atresia and to compare them with healthy controls and 2) associated motility and permeability changes. In addition, we analyzed the ENS neurochemical coding of myenteric plexus from human neonatal atresia samples.

## Results

### A. Experimental Atresia

#### A.1 Phenotypical studies

Only rat fetuses with successful prenatal obstruction (n = 44) were included in the study. Birth weight was 4.5±0.5 g and total small bowel length was 12.8±1.3 cm. Littermate birth weight was 5.3±0.4 g (n = 12; p = 0.005) and intestinal length 15.3±1.8 cm (n = 12; p = 0.002).

**Figure 1:**
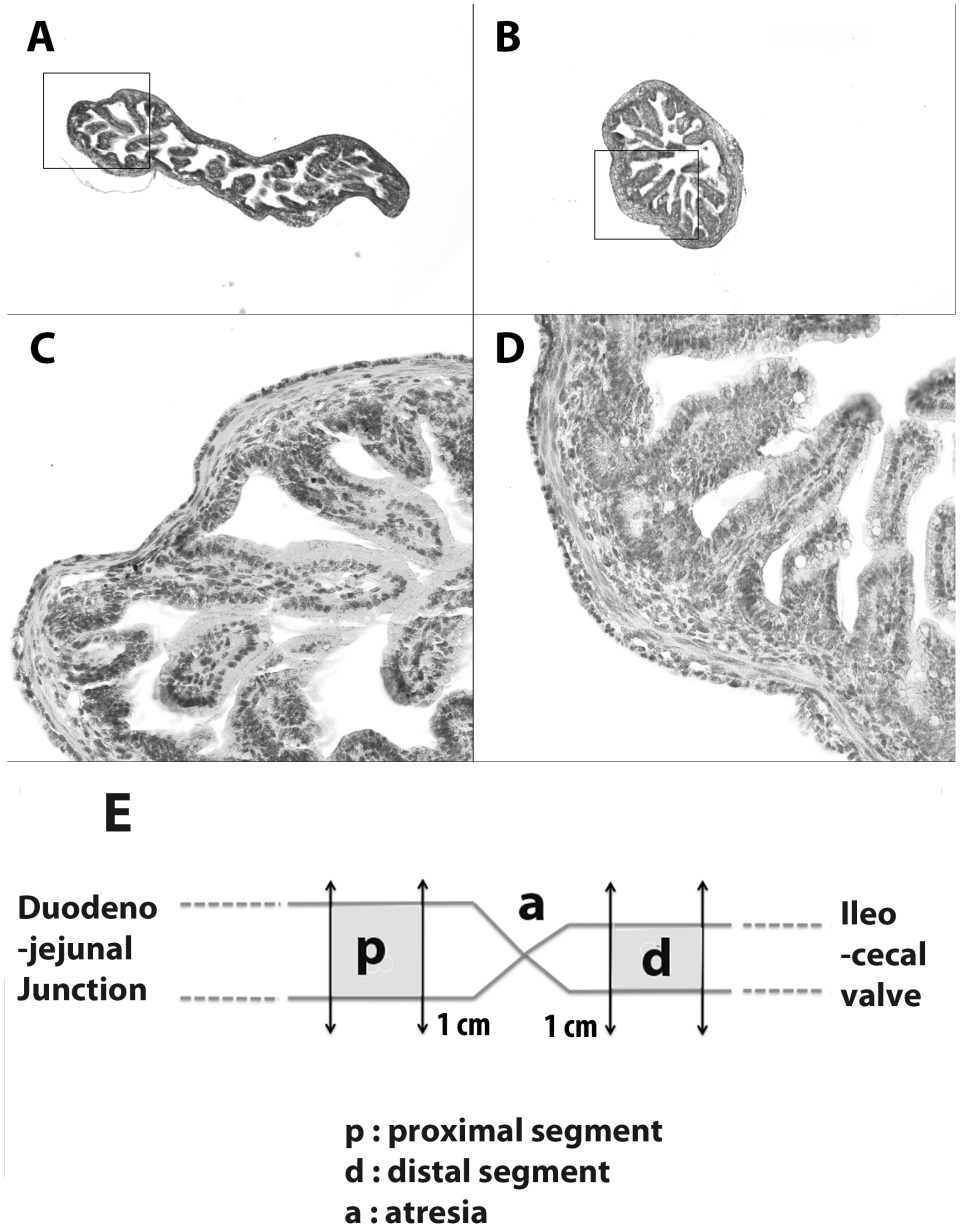
Histology of intestinal segments in experimental atresia. The external diameter of the gut was 1.5 to 2.2 times larger in the segment proximal to the atresia (**A**) than in the distal segment (**B**). Magnifications 5X. No difference in epithelial organization or inflammatory cell infiltration was observed between proximal (**C**) and distal (**D**) segments. Magnifications 25X. (**E**) For each experiment, proximal segments were taken 1 cm immediately above the atresia and distal segments 1 cm immediately below the atresia.

The atresia was located at a median of 8 cm (range 2–13 cm) from the duodeno-jejunal junction. The external diameter of the gut was significantly larger (1.8±0.4-fold) in the segments proximal to the atresia than in the distal segments (figure 1A,B,C,D). For each experiment, the proximal segment was defined as the intestinal sample taken 1 cm immediately above the atresia and the distal segment as the intestinal sample taken 1 cm immediately below it (figure 1E).

**Figure 2:**
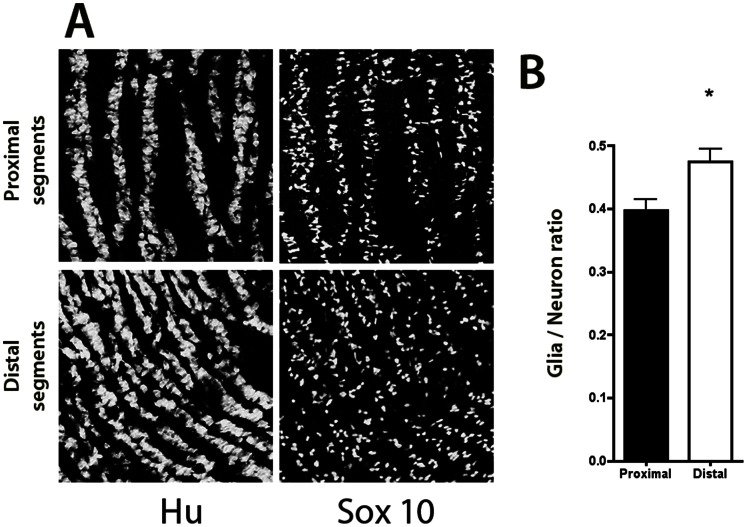
Neurochemical plasticity of the myenteric plexus in experimental intestinal atresia. The density of Hu-IR neurons was significantly higher in distal than in proximal segments whereas the density of SOX10-IR glial cells was similar in the two segments (**A**), resulting in a significantly higher glia/neurons ratio in the distal segment (n = 12; *p<0.05; two-tailed Wilcoxon test). (**B**). Magnification 40X.

The density of Hu-IR neurons (expressed as a function of tissue area (microm^2^)) was significantly lower in the proximal segments than in the distal segments (29.7±5.4% vs 38.6±0.8% respectively, n = 12; p = 0.004; figure 2A). Interestingly, neuronal surface area in the proximal segments was significantly larger than in the distal segments (62±7 microm^2^ vs 48, ±10 microm^2^ respectively, n = 12; p = 0.004). There was no significant difference in the density of SOX10-IR glial cells (12.5±0.2% vs 16.7±6.1% respectively, n = 12; p = 0.11; figure 2A). However, the glia/neuron ratio was significantly lower above than below the atresia (39.6±6.7% vs 46.7±6.5% respectively, p = 0.02; figure 2B). Caspase-3 staining showed no significant difference between the two segments (data not shown).

**Figure 3:**
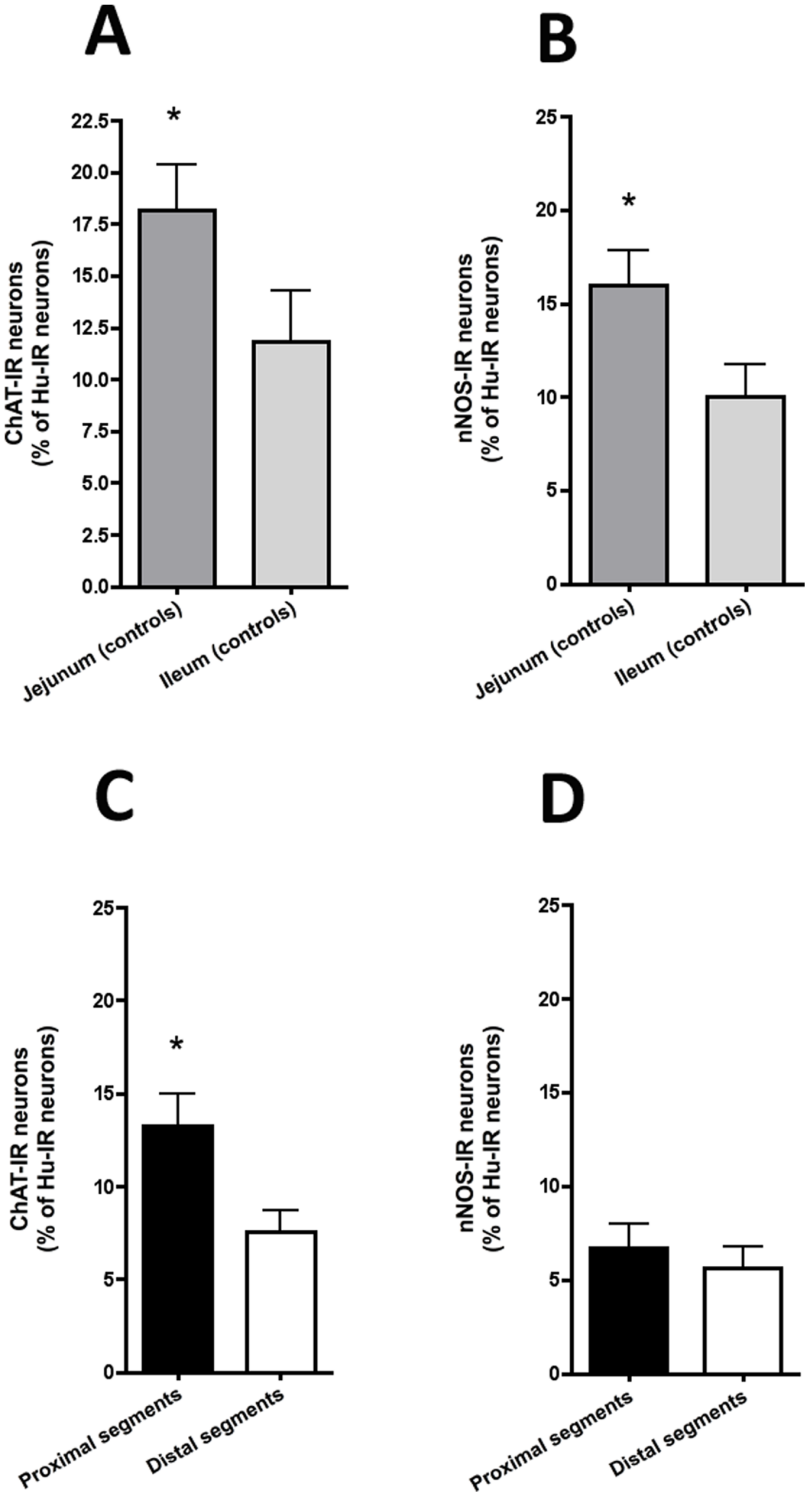
Neuronal phenotype of the myenteric plexus in experimental intestinal atresia and controls. (**A**) In controls, the proportions of ChAT-IR myenteric neurons (normalized to the total number of Hu-IR neurons) was significantly higher in jejunal than in ileal segments (n = 10; *p<0.05; two-tailed Wilcoxon test). (**B**) The proportion of nNOS–IR myenteric neurons (normalized to the total number of Hu-IR neurons) was significantly higher in jejunal than in ileal segments (n = 10; *p<0.05; two-tailed Wilcoxon test). (**C**) In atresia, the proportion of ChAT-IR myenteric neurons (normalized to the total number of Hu-IR neurons) was significantly higher in proximal than in distal segments (n = 12; *p<0.05; two-tailed Wilcoxon test). (**D**) In contrast, the proportion of nNOS–IR myenteric neurons (normalized to the total number of Hu-IR neurons) was similar in proximal and distal segments (n = 12; two-tailed Wilcoxon test).

We then examined the neurochemical phenotype of the myenteric neurons in atresia and in jejunum and ileum of controls. In control animals, the proportion of ChAT-IR neurons normalized to total number of Hu-IR neurons was significantly higher in jejunum as compared to ileum (18.2±7.6% vs. 11.8±5.4% respectively, n = 12; p = 0.04, figure 3A). In atresia, the proportion of ChAT-IR neurons in proximal segments was significantly higher than in the distal segments (13.2±6.2% vs 7.5±4.3% respectively, n = 12; p = 0.005; figure 3C). When compared to controls, the proportion of ChAT-IR neurons in proximal segments of atresia was not significantly different from both jejunum and ileum controls. In contrast, in distal segments the proportion of ChAT-IR neurons was significantly lower to the one observed in jejunum but not ileum of controls (p = 0.001 and p = 0.2 respectively).

In control animals, the proportion of nNOS-IR neurons was significantly higher in jejunum as compared to ileum (16.2±9.5% vs. 6.4±5.7% respectively, n = 12; p = 0.01, figure 3B). In atresia, the proportion of nNOS-IR neurons was similar in proximal and distal segments (6.7±4.6% vs 5.6±4.2% respectively, p = 0.25; figure 3D). When compared to controls, the proportion of nNOS-IR neurons in proximal segments of atresia was significantly lower to the control jejunum (p<0.01) but not ileum. In distal segments of atresia the proportion of nNOS-IR neurons was similar to the controls (both jejunum and ileum).

#### A2. Functional studies

We then examined whether ENS phenotypic changes observed after prenatal intestinal atresia were associated with functional changes.

Under basal condition, no detectable spontaneous contractile activity occurred in neither proximal, distal nor controls longitudinal muscle strips.

**Figure 4:**
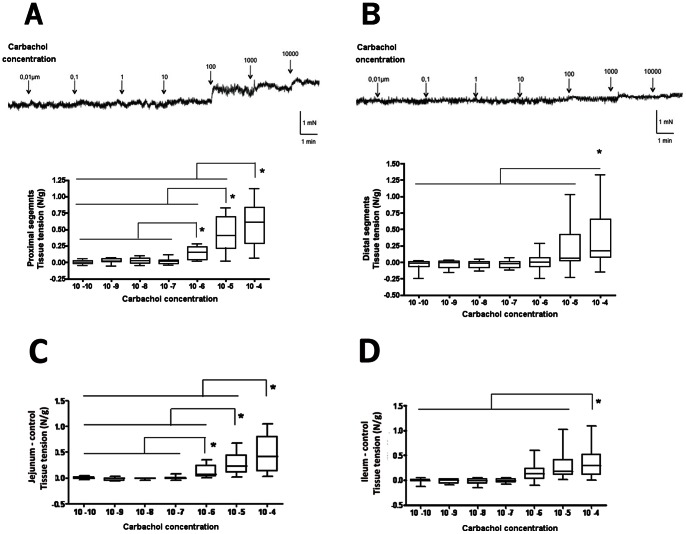
Carbachol-induced contraction in experimental intestinal atresia. (**A**) Typical recording of carbachol dose-response stimulation of contraction in proximal segment (Upper Panel). Proximal segments showed a significant increase in the contractile response starting at 1 µM carbachol (Lower Panel). (**B**) Typical recording of carbachol dose-response stimulation of contraction in distal segment from the same atresia as in (A) (Upper Panel). Distal segments showed a significant increase in the contractile response starting only at 100 µM carbachol (Lower Panel). n = 8; *p<0.05 as compared to 1 nM carbachol; Kruskal-Wallis analysis of variance. Data are medians, 5–95th percentiles and range. In controls, jejunal segments showed a significant increase in the contractile response starting at 1 µM carbachol (**C**), whereas ileal segments showed a significant increase in the contractile response starting only at 100 µM carbachol (**D**). n = 8; *p<0.05 as compared to 1 nM carbachol; Kruskal-Wallis analysis of variance. Data are medians, 5–95th percentiles and range.

The contractile response of longitudinal muscle was assessed ex vivo in organ chambers in atresia samples (n = 8) and controls samples (n = 8). Carbachol induced a concentration-dependent contractile response in proximal segments, starting at a concentration of 1 microM (figure 4A). This response was similar to the one observed in the jejunum of controls (figure 4C). In contrast, the concentration required to obtain a dose-dependant contractile response in distal segments was 100-fold higher, at 100 microM (figure 4B), which was comparable to response observed in the ileum of controls (figure 4D).

**Figure 5:**
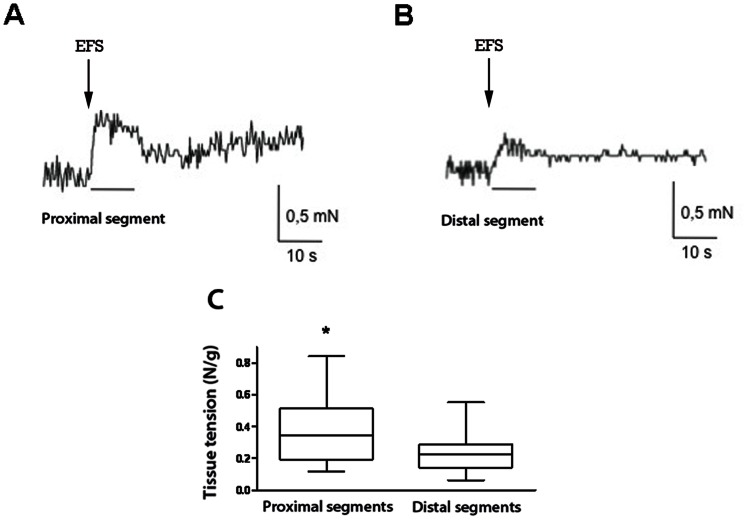
Neurally mediated contractile responses of ileal longitudinal muscle strips to electrical field stimulation (EFS) in experimental intestinal atresia. (**A**) Typical recording after EFS in proximal segment showing an increase in the contractile response. (**B**) Typical recording after EFS in distal segment from the same atresia as in (A) showing a lower contractile response than in proximal segment. (**C**) Neurally mediated contraction induced by EFS of longitudinal muscle was significantly higher in proximal than in distal segments (n = 12; *p<0.01; paired *t* test). Data are medians, 5–95th percentiles and range.

The neurally mediated contractile response of small-intestine longitudinal muscle was also assessed ex vivo in organ chambers in atresia samples (n = 12). EFS-induced contractile responses were 1.6-fold higher in proximal than distal atresia segments (p = 0.007, figure 5).

**Figure 6:**
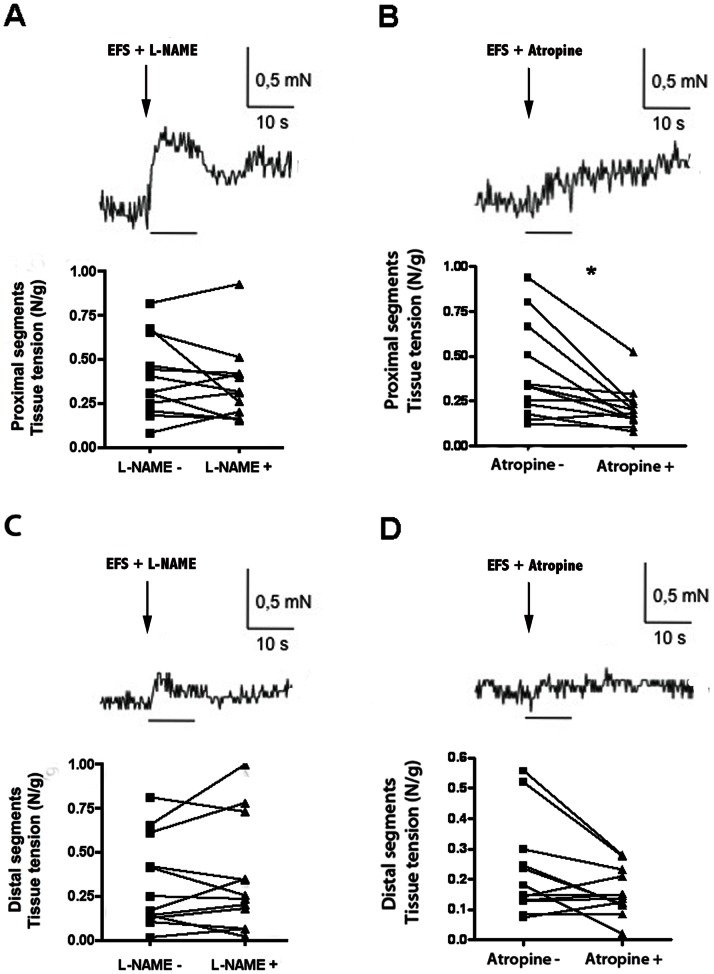
EFS-induced induced area under the curve (AUC) in absence or in presence of *N*-nitro-l-arginine methyl ester (l-NAME) and atropine in experimental intestinal atresia. Pre-treatment with N-nitro-L-arginine ester (L-NAME 50 µM) did not affect the EFS-induced contractile response of proximal segments (**A**) when compared to EFS-induced contractile response alone (see figure 4A), while atropine (1 µM) significantly reduced the EFS-induced contractile response of the same segments (**B**) when compared to EFS-induced contractile response alone (see figure 4B) (n = 12; *p = 0.002, two-tailed Wilcoxon test). Pre-treatment with L-Name (50 µM) (**C**) or atropine (1 µM) (**D**) did not affect the EFS-induced contractile response of distal segments (n = 12; p = 0.15, two-tailed Wilcoxon test). Data are individual values.

L-NAME (N-nitro-L-arginine methyl esther; 5×10^−4^ mol/l) did not significantly modify EFS-induced contractile responses in neither proximal nor distal segments (figures 6A,C). However, contractile responses in proximal segments were inhibited by atropine, whereas atropine had no significant effect on the contractile response of distal segments (p = 0.002 and p = 0.15, respectively; figures 6B,D).

Tissue resistance was not significantly different between proximal (15.1±4.3 ohms.cm^−2^; n = 12) and distal segments (16.9±5.1 ohms.cm^−2^) or between atresia segments and control jejunum (18.2±3.5 ohms. cm^−2^) or control ileum (16.8±7.3 ohms. cm^−2^).

**Figure 7:**
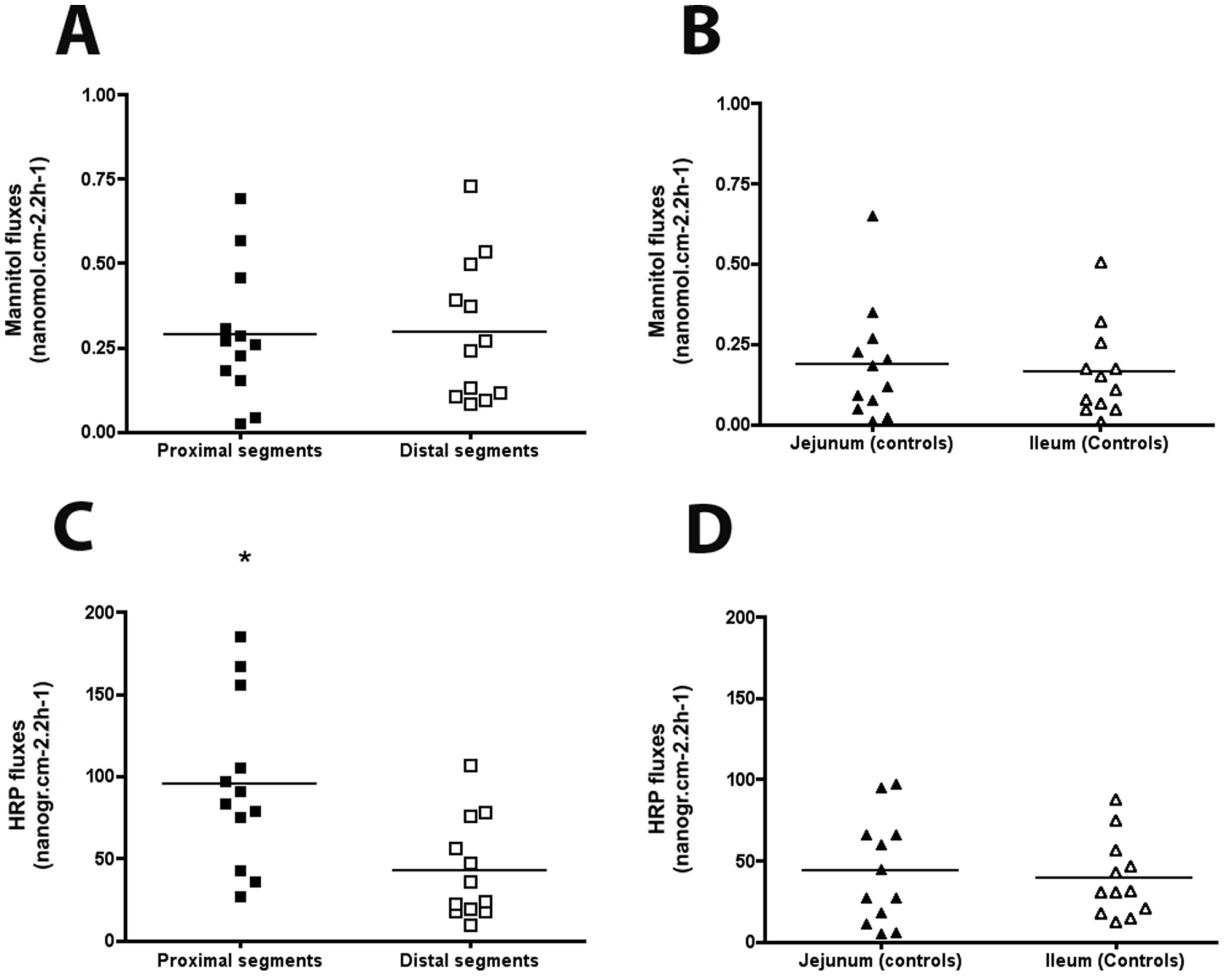
Mannitol and horseradish peroxidase (HRP) permeability in experimental intestinal atresia. (**A**) In atresia, ^14^C-mannitol flux was similar in proximal and distal segments. (**B**) In control animals, ^14^C-mannitol flux was similar in the jejunum and distal ileum. (**C**) In atresia, HRP flux was significantly higher in the proximal than distal segments. (**D**) In controls, HRP flux was similar in the jejunum and distal ileum (n = 12; *p<0.05; two-tailed Wilcoxon test). Data are individual values, the mean being represented by a bar.

No significant difference was observed in mannitol flux between the proximal and distal segments (289±20 nmol.cm^−2^.2 h^−1^ and 296±21 nmol.cm^−2^.2 h^−1^ respectively, figure 7A) or between jejunal and ileal controls (188±18 nmol.cm^−2^.2 h^−1^ and 163±14.cm^−2^.2 h^−1^ respectively; figure 7B).

In contrast, in atresia, HRP (horseradish peroxidase) flux was significantly higher in the proximal segments than in the distal segments (102±56 ng.cm^−2^.2 h^−1^ vs 36±30 ng.cm^−2^.2 h^−1^, p = 0.002, figure 7C). HRP fluxes in proximal but not distal segments were higher to jejunal segments (43±31 ng.cm^−2^.2 h^−1^, p = 0.01) and ileal segments (45±31 ng.cm^−2^.2 h^−1^, p = 0.009) of controls (figure 7D).

#### A3. Cytokine studies

We then examined whether functional changes were associated with variations in proinflammatory cytokines expression. Compared to distal segments, mRNA levels in the proximal segments were 9.5-fold higher for Cxcl2 (p = 0.002), 2.7-fold higher for Il6 (p = 0.01), 1.7-fold higher for Il10 (p = 0.01) and 1.6-fold higher for Il1 beta (p = 0.03). In contrast, iNos and Ifn gamma mRNA expressions were similar in the two segments (p = 0.77 and p = 0.06 respectively).

### B. Human Atresia

#### B1. Intestinal morphology

The human atresia (N = 8) was located at a median of 51 cm (range 3–125 cm) from the duodeno-jejunal junction. The external diameters ranged from 11 to 25 mm for the proximal segments and from 4 to 6 mm for the distal segments. The ratio of the diameters was 3.6±1.3.

**Figure 8:**
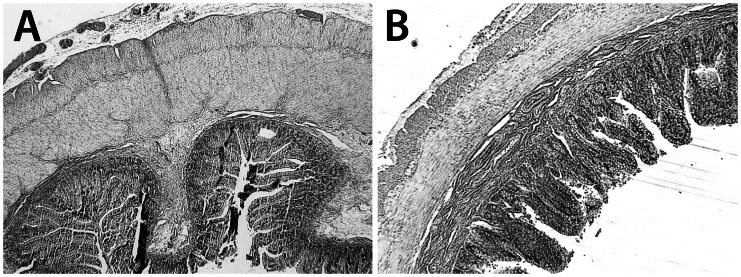
H&S histology of intestinal segments in human atresia. In proximal (**A**) and distal segments (**B**) of human atresia the mucosa had a normal aspect, with no alterations of the villi or crypts, but mild infiltration of the submucosa. Muscle hypertrophy was present in the proximal segments, predominating in the internal circular layer (**A**). Magnification ×25.

H&S staining showed a normal mucosa in all cases, with submucosal edema in five cases and mucosal hemorrhagic spots in three cases. In all cases, muscular hypertrophy was present in the proximal segments when compared to the distal segments, with a muscle thickness ratio of 1.4 to 2.2 (figure 8). As previously reported, the myenteric plexus in the proximal segment appeared as spaced knots, whereas it had a more continuous aspect in the distal segment [16].

#### B2. Phenotypical studies

**Figure 9:**
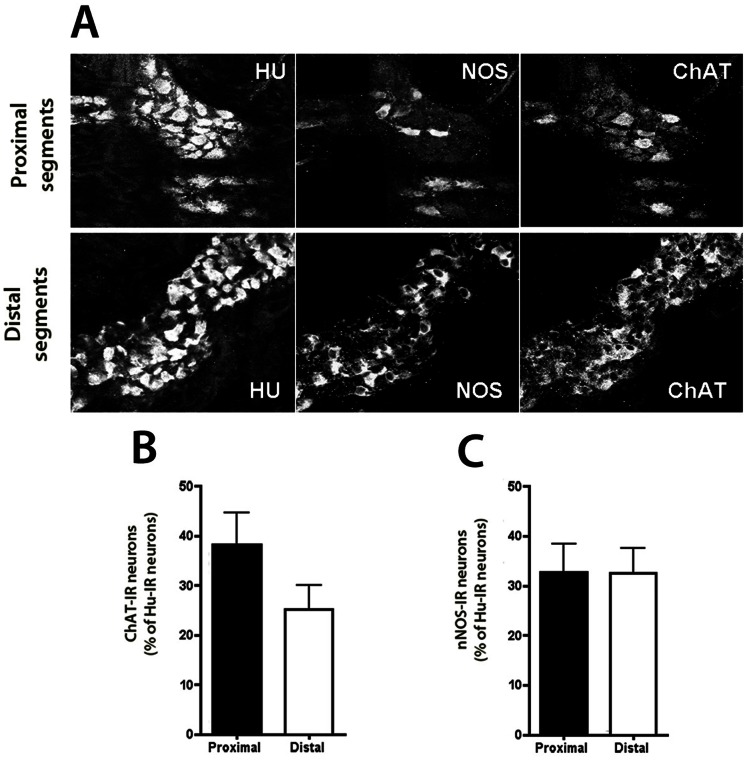
Neurochemical plasticity of intestinal segments in human atresia. (**A,B**) The proportion of ChAT-IR myenteric neurons (normalized to the total number of Hu-IR neurons) was slightly but not significantly lower in distal segments than in proximal segments (n = 8; p = 0.31; two-tailed Wilcoxon test). (**A,C**) The proportion of nitric oxide synthase nNOS-IR neurons (normalized to the total number of Hu-IR neurons) was similar in proximal and distal segments (n = 8; p = 0,84; two-tailed Wilcoxon test). Magnifications 40X.

In human atresia, the proportion of ChAT-IR myenteric neurons was higher in the proximal (38±16%) than distal segments (25±12%), although the difference did not reach statistical significance (n = 8; p = 0.31; figures 9A,B) whereas ChAT mRNA expression was significantly higher in the proximal than distal segment (2.5-fold, p<0.05).

The proportion of nNOS-IR myenteric neurons was similar in the proximal and distal segments (33±14% vs 32±12% respectively, p = 0.84; figures 9A,C) and nNos mRNA levels were similar in the proximal and distal segments.

#### B3. Cytokine expression

No difference in the mRNA expression of cytokines CXCL2 (p = 0.7), IL1 beta (p = 0.7), IL6 (p = 0.9), IL-10 (p = 0.8), iNOS (p = 0.8) or IFN gamma (p = 0.4) was observed (data not shown).

## Materials and Methods

### Ethics Statement

The experimental protocol was approved by the French animal care and use committee (Comite National de Reflexion Ethique sur l’Experimentation Animale N° P2.SS.025.07) and all animal procedures were performed according to the French guidelines for animal protection and animal welfare.

According to the guidelines of French Ethics Committee for research on human tissues and after Institutional Review Board CPP Necker approval, atresia specimens were considered as “residual tissues”. Parents’ written informed consent was obtained for all the patients and the tissue collection was declared to the Research Ministry N° DC-2009-955.

### A. Intestinal Samples Origin

#### A1. Animal model

Time-dated pregnant rats were purchased from Janvier Laboratories (Le Genest Saint Isle, France). The surgical procedure was performed on day 18 of gestation (term: 22 days±1), as previously described [17]. Briefly, general anesthesia was induced in pregnant female Wistar rats by intramuscular injection of ketamine (12 mg/100 g body weight) and chlorpromazine (0.07 mg/100 g). After maternal hysterotomy the fetal wall was incised and sterile saline was injected to extrude a bowel loop, which was ligated with 11-0 polypropylene and pushed back into the fetal abdomen. Laparotomy and hysterotomy were then closed using respectively 9-0 and 8-0 polypropylene. The maternal laparotomy was closed with 2 layers of 4-0 polypropylene running sutures.

Caesarean delivery was performed on day 21 of gestation. The fetuses were killed according to the guidelines of the animal care and use committee and the whole intestine was removed. Atresia was considered successful if the fetus was alive at delivery and if a stricture of the small intestinal was clearly visible, together with a difference in gut diameter above and below the ligation, and a difference in the color of the two segments (green bilious content in the proximal segment contrasting with an uncolored distal segment).

We compared two segments from each animal; one located 1 cm immediately above the atresia (“proximal segment”) and the other 1 cm immediately below it (“distal segment”). In addition, segments of intestine from rat pups obtained after caesarian delivery at day 21 were collected and processed similarly to atresia segments. Two different parts were studied: jejunal samples, located at one cm below the duodeno-pancreatic zone and ileal samples, located at one cm before the ileocaecal valve.

#### A2. Human atresia

We studied eight neonates (6 girls and 2 boys) born with isolated intestinal atresia with a median gestational age of 36 weeks (range 32–39) and mean birth weight of 2660±540 g. In keeping with French ethical guidelines on research involving human tissues, intestinal fragments resected during curative surgery at birth were considered as residual tissue. Intestinal dilation had been diagnosed prenatally in all but one of the children, at a median of 27 weeks of gestation (range 22–33). None of the patients had short bowel syndrome but all showed post-operative intestinal dysmotility with a median duration of parenteral nutrition of 36 days (range 22–136 days). The median length of hospital stay was 54 days (range 24–141 days).

Because of the small size of the human atresia distal segment samples, ex vivo functional studies could not be performed and the priority for IHC staining and PCR analysis was given.

### B. Samples Analyses

#### B1. Phenotype

Intestinal samples were fixed overnight at 4°C in 0.1 mol/l PBS containing 4% paraformaldehyde. Whole mounts of the longitudinal muscle myenteric plexus were prepared by microdissection. After permeabilisation for 3 h in PBS/NaN_3_ containing 0.5% Triton X-100 and 5% horse serum (Sigma), whole-mount preparations of rat and human atresia were incubated overnight with anti-choline acetyltransferase, anti-neuronal nitric oxide synthase and anti-Hu antibodies. Anti-Sox10 and anti-Caspase3 antibodies were only used for rat specimens. Glial cells were counted after Sox10 labeling. Apoptosis was analyzed after Caspase3 staining. The tissue samples were then washed with PBS (3×10 min) and incubated for 12 h with the following secondary antibodies. Antibody sources and concentrations are shown in Table S1.

Specimens were examined with LSM 510 META confocal fluorescence microscope equipped with a 40X objective (Carl Zeiss AG, Oberkochen, Germany). The proportion of ChAT-immunoreactive (IR) and nNOS-IR neurons was normalized to the total number of Hu-IR neurons. Contiguous non-overlapping fields were analyzed until 500 Hu-IR neurons had been counted. The packing densities of glia and neurons were also evaluated and expressed as a function of tissue area (µm^2^), as previously described [18]. The glia/neuron ratio was calculated. Quantification was performed with Image J software (National Institutes of Health, Bethesda, Maryland, USA). The individual surface area of Hu-IR neurons was also measured. For each intestinal sample, the value used was the mean of 100 neurons in a X40 magnification view and expressed in µm^2^ unit.

Whole-thickness intestinal segments were analyzed by real-time PCR. Total RNA was extracted with Rneasy Plus minikits (Qiagen) according to the manufacturer’s instructions, and transcripts were analyzed with Taqman assays (Applied Biosystem) on a 7300 Applied Biosystems device. Cytokine (CxCl2, Il1 beta, Il6, Il10, Ifn gamma and iNos) were normalized to S6. ChAT and nNos transcripts were normalized to Pgp9.5. Cytokine Results were analyzed with the delta Ct method. Primer sources are shown in Table S2.

#### B2. Function

The contractile activity of intestinal segments was analyzed as previously described [19]. Briefly, longitudinal muscle intestinal segments 1 cm long were suspended under 0.2 N of tension in a 7-ml organ bath containing oxygenated (5% CO_2_, 95% O_2_) Krebs-bicarbonate solution (in mM: 117 NaCl, 4.7 KCl, 1.2 MgCl_6_H_2_O, 11.2 NaH_2_PO_4_, 25 NaHCO_3_, 2.5 CaCl_2_, 2 H_2_O, 11 glucose), and allowed to equilibrate for 30 minutes. Isometric contractions were recorded with a force transducer (Ugo Basile N°7005; Comerio, VA, Italy). Electrical field stimulation (EFS) was applied with a stimulator connected to platinum electrodes in order to activate enteric neurons and to characterize neurally mediated contractile responses. The parameters of stimulation were as follows: 12 V, 20 Hz, train duration 10 s and stimulus pulse duration 300 micros. The results were normalized to tissue-wet weight and expressed in N/g.

The neuropharmacology of EFS-induced contractile responses was analyzed in the presence of a muscarinic antagonist (atropine sulfate 10^−6^ mol/l; Sigma) and a nitric oxide synthase inhibitor (N^G^-nitro-L-arginine methyl ester; L-NAME 5×10^−4^ mol/l; Sigma). Atropine and L-NAME were applied 15 minutes before EFS. At the end of the experiments, after abundant washing for 30 minutes, increasing concentrations of the cholinergic agonist carbachol chloride (10^−11^ to 10^−4^ mol/l, Sigma) were added to evaluate contractile responses.

Epithelial barrier permeability was analyzed on intestinal samples as previously described [20], [21]. Full-thickness intestinal segments were mounted in Ussing chambers, exposing 0.35 cm2 of tissue to Krebs buffer-bicarbonate solution maintained at 37°C.

Horseradish peroxidase (HRP, 44 kDa) was used to analyze changes in transcellular permeability. Type VI HRP (10^–5^ M, Sigma Chemical Co, St Louis, MO, USA) was added to the luminal buffer 15 min after sample mounting. After 2 hours, the enzymatic activity of intact HRP was measured with a modified Worthington method.

^14^C-mannitol (182 Da, 10^–9^ mmol/l; specific activity: 20 Ci/mmol; ARC, Missouri, USA) was used to analyze changes in paracellular permeability. ^14^C-mannitol was added to the mucosal side. After 2 hours, ^14^C-mannitol fluxes (mucosal-to-serosal) were analyzed with a liquid scintillation counter (Tri-Carb 2100TR; Packard, USA).

### Statistical Analysis

Non-parametric tests were used. Results for proximal and distal segments were compared by using Wilcoxon’s paired two-tailed test or the Kruskal-Wallis test. Differences were considered significant at p<0.05.

## Discussion

In our experimental model of prenatal intestinal obstruction, prenatal mechanical constraints resulted in major phenotypic and functional differences between the myenteric plexus immediately proximal and distal to the obstacle, whereas these two adjacent segments were expected to be similar. These changes were characterized by a higher proportion of cholinergic neurons and an increased cholinergic neuromuscular contractile response in the proximal segments as compared to the distal segments.

The rat model of prenatal obstruction was developed to determine whether and how prenatal atresia might affect the functional and/or phenotypic properties of the upstream and downstream enteric nervous system (ENS). In this model, complete intestinal obstruction is induced prenatally, resulting in dilation of the upstream segment and narrowing of the downstream segment, as observed in other experimental models and in human prenatal atresia [11], [13], [22]. Like in human neonates, rat pups with intestinal atresia had a low birth weight, partly attributed to the lack of amniotic fluid circulation and absorption in the distal intestine [23], [24]. One noteworthy difference between the experimental model and the clinical setting is the duration of the obstruction. Indeed, experimental obstruction was induced three days before birth in rats, while antenatal occlusion usually occurs during the second trimester in humans. This may explain why increased cytokines mRNA expression was observed in rat intestinal samples but not in human neonates, in whom a fibrous scar has had time to form before birth [25], [26].

A major finding in our study is the identification of neuroplastic changes in the ENS between the segments immediately upstream and downstream of the atresia. We observed a significant reduction in neuronal density in the proximal segments. This difference could result from tissue distension induced by the obstruction. This mechanism has been suggested in an adult rat model of occlusion, where a lower frequency of nerve fibers in the hypertrophic proximal segment was also observed [27]. Alternatively, as no difference was observed in caspase-3 staining between the two segments, our observation might be compared to what is observed during antenatal ENS maturation, where the reduced density of myenteric neurons was attributed to gut architectural changes rather than to active cell death [28].

Besides changes in neuronal density, we observed a higher proportion of ChAT-IR neurons in the proximal segments, as compared to the distal segments. As post-natal maturation of the ENS has been associated with an age-dependent increase in the expression of the vesicular acetylcholine transporter and ChAT in myenteric neurons [29], [30], these changes might reflect a lower maturation state of the ENS in the distal part of the segment as compared to the proximal one. Phenotypic differences were observed in control jejunum and ileum, consistent with the known rostro-caudal gradient of ENS maturation. As the “proximal” and “distal” atresia segments were almost adjacent, no difference might have been expected. The proportion of ChAT-IR neurons in the atresia distal segment (with a median location at midgut) was however significantly lower than in control jejunum and lower, and although not significant than in control ileum.

Postnatal maturation of ENS has been associated with increased in neuronal surface [29], [31]. Further supporting a lack of maturation in distal segment vs. proximal one is our observation that neuronal surface area is significantly reduced in distal as compared to proximal segment. Whereas a rostrocaudal gradient was also suggested by the differences in the proportion of nNOS-IR neurons in controls, no difference was observed in atresia segments for this marker. Phenotypic studies in human atresia were comparable to the one observed in the experimental model, sustaining the higher proportion of ChAT-IR neurons in proximal segments.

Intestinal motor responses to carbachol also differed between the two segments of atresia. In particular, the proximal segments exhibited a stronger contractile response to carbachol as compared to the distal one. Furthermore, carbachol induced dose-responses in the proximal and the distal segment were similar to the one observed in the jejunum and ileum of healthy controls, respectively. This further reinforces the hypothesis of a defect in functional maturity of the distal segment. In controls, the lower contractile response induced by carbachol observed in the ileum vs. jejunum is consistent with the rostro caudal maturation gradient of the gut motor activity previously reported [32]. In contrast no difference was observed in the response to L-Name between proximal and distal segments of atresia, which was consistent with the absence in difference in nNOS phenotype. Altogether, these results suggest that contractility but not relaxation is affected by atresia.

Concerning epithelial barrier function, transcellular permeability to HRP was significantly higher in the proximal segment than distal, and was also higher than in segments from controls. The mechanism underlying this increased permeability is unclear. IFN gamma has been shown to enhance paracellular and transcellular permeability [33], [34] but in our model, paracellular permeability and Ifn gamma mRNA levels were similar between the proximal and distal segments. Alternatively, the increased proportion of cholinergic neurons observed in proximal segments could account for enhanced intestinal permeability, as acetylcholine is known to increase transcellular intestinal permeability [35]–[37]. As changes in transcellular transport could promote bacterial translocation, the increased transcytosis above the atresia observed in our experimental model, and also compared to healthy controls, provides pathophysiological support to gut-derived sepsis observed after surgical treatment of intestinal atresia [38], although these permeability experiments must be repeated in human atresia.

The mechanisms responsible for the changes observed after prenatal intestinal obstruction are unclear, but they might result in part from a lack of mechanical or nutritional stimulation in the distal segment, that is mandatory for phenotypic and functional maturation. Recent studies have shown that activity-dependent and nutrition-dependent mechanisms can participate in the regulation of neuromediator expression in the ENS in embryonic neurons [39]. Atresia results in an interruption of amniotic fluid flow below the obstruction, and the observed phenotypic changes could thus also be a direct consequence of the intestinal obstacle. Amniotic fluid has indeed been reported to contain various neurotrophic factors that participate in the maturation of gut functions [40] and could enhance ENS maturation by activating enteric neurons, leading to increased expression of enteric neuromediators [29], [41]. Another possible explanation for this difference is that atresia might disrupt a physiological rostro-caudal intestinal maturation gradient. Indeed, gestational evolution of small intestine motility along the gut has been reported in preterm and term infants [42] and ENS development have been described within the rat colon during the postnatal period [29].

In order to determine if the observations made in the rat model were consistent with the human disorder, phenotypic studies were performed on 8 intestinal samples from babies with atresia and post-operative intestinal dysmotility (parenteral nutrition duration from 22 to 136 days). Analysis showed neuronal changes between proximal and distal segments similar to those observed in the experimental model. Because the experimental model reproduces the macroscopic findings observed in human atresia, one might expect similar functional findings in humans, although studies of a larger cohort of patients, including a correlation analysis of intestinal motility, are needed to confirm this important point.

In conclusion, our findings point to functional and phenotypical changes in both parts of intestinal atresia, whereas these adjacent segments were expected to be comparable. Reduced ChAT expression by myenteric neurons and a reduced contractile response in the intestinal distal segment of the atresia may contribute to the intestinal motility disorders frequently observed after surgical treatment of congenital atresia. Increased transcellular permeability in proximal segments could also contribute to increased risk of bacterial translocation in these patients. Therefore, analysis of phenotypical and functional abnormalities in neonates with intestinal atresia might help to identify markers predictive of the severity of the alterations and to define guidelines for optimal postoperative management.

## Supporting Information

Table S1 Antibody references and concentrations for rat and human immunofluorescence analysis.
(DOC)

Table S2 TaqMan oligonucleotide primers used for rat and human PCR analysis.
(DOC)
