# Supplementary material for: Prenatal Intestinal Obstruction Affects the Myenteric Plexus and Causes Functional Bowel Impairment in Fetal Rat Experimental Model of Intestinal Atresia
Source: PLoS One. 2013 May 8;8(5):e62292. doi: 10.1371/journal.pone.0062292 (PMC3648556; doi:10.1371/journal.pone.0062292)
Supplement: Table S1 — Antibody references and concentrations for rat and human immunofluorescence analysis. (DOC) [file pone.0062292.s001.doc]

**Table S1. Antibody references and concentrations for rat and human immunofluorescence analysis**

| **Primary antibody** |  | **Species** | **Dilution** |
| --- | --- | --- | --- |
| Choline Acetyltransferase | Chemicon Intenational | Goat | 1:200 |
| Neuronal nitric oxide synthase | Alexis Biochemicals | Rabbit | 1:2000 |
| Hu | Molecular Probes | Mouse | 1:200 |
| Sox 10 | Santa Cruz Biotechnology | Goat | 1:500 |
| Caspase 3 | Sigma | Rabbit | 1:1000 |
|  |  |  |  |
| **Secondary antibody** |  |  |  |
| CY3 | Jackson Immunoresearch | Donkey | 1:500 |
| CY5 | Jackson Immunoresearch | Goat | 1:500 |
| FluoProbes 488 | Interchim | Donkey | 1:200 |
