# Supplementary material for: Prenatal Intestinal Obstruction Affects the Myenteric Plexus and Causes Functional Bowel Impairment in Fetal Rat Experimental Model of Intestinal Atresia
Source: PLoS One. 2013 May 8;8(5):e62292. doi: 10.1371/journal.pone.0062292 (PMC3648556; doi:10.1371/journal.pone.0062292)
Supplement: Table S2 — TaqMan oligonucleotide primers used for rat and human PCR analysis. (DOC) [file pone.0062292.s002.doc]

**Table S2. TaqMan oligonucleotide primers used for rat and human PCR analysis**

|  | **Gene** | **Code** | **Primer Reference** | **Size** |
| --- | --- | --- | --- | --- |
| **RAT** | Cxcl2 | NM_053647.1 | Rn00586403_m1 | 85 bp |
|  | Ifnγ | NM_138880.2 | Rn99999014_m1 | 95 bp |
|  | Il1β | NM_031512.2 | Rn00580432_m1 | 74 bp |
|  | Il6 | NM_012589.1 | Rn99999011_m1 | 90 bp |
|  | Il10 | NM_012854.2 | Rn00563409_m1 | 70 bp |
|  | iNos | NM_012611.3 | Rn00561646_m1 | 77bp |
|  | Rps6Ka4 | NM_001108517.1 | Rn01760381_m1 | 104 bp |
|  |  |  |  |  |
| **HUMAN** | Chat | [NM_001142929.1](http://www.ncbi.nlm.nih.gov/entrez/viewer.fcgi?val=NM_001142929.1#_blank) | Hs00252848_m1 | 64 bp |
|  | Cxcl2 | [NM_002089.3](http://www.ncbi.nlm.nih.gov/entrez/viewer.fcgi?val=NM_002089.3#_blank) | Hs00601975_m1 | 100 bp |
|  | Ifnγ | NM_000619.2 | Hs99999041_m1 | 117 bp |
|  | Il1β | [NM_000576.2](http://www.ncbi.nlm.nih.gov/entrez/viewer.fcgi?val=NM_000576.2#_blank) | Hs00174097_m1 | 94 bp |
|  | Il6 | [NM_000600.2](http://www.ncbi.nlm.nih.gov/entrez/viewer.fcgi?val=NM_000600.2#_blank) | Hs00174131_m1 | 95 bp |
|  | Il10 | [NM_000572.2](http://www.ncbi.nlm.nih.gov/entrez/viewer.fcgi?val=NM_000572.2#_blank) | Hs99999035_m1 | 86 bp |
|  | iNos | NM_000625.4 | Hs01075529_m1 | 67 bp |
|  | nNos | [NM_000620.2](http://www.ncbi.nlm.nih.gov/entrez/viewer.fcgi?val=NM_000620.2#_blank) | Hs00167223_m1 | 61 bp |
|  | Pgp9.5 | [NM_004181.3](http://www.ncbi.nlm.nih.gov/entrez/viewer.fcgi?val=NM_004181.3#_blank) | Hs00188233_m1 | 100 bp |
|  | S6 | [NM_153001.1](http://www.ncbi.nlm.nih.gov/entrez/viewer.fcgi?val=NM_153001.1#_blank) | Hs00197826_m1 | 83 bp |
